# Alcohol Use and Abuse Conspires With HIV Infection to Aggravate Intestinal Dysbiosis and Increase Microbial Translocation in People Living With HIV: A Review

**DOI:** 10.3389/fimmu.2021.741658

**Published:** 2021-12-17

**Authors:** Jiangyu Yan, Jing Ouyang, Stéphane Isnard, Xin Zhou, Vijay Harypursat, Jean-Pierre Routy, Yaokai Chen

**Affiliations:** ^1^ Clinical Research Center, Chongqing Public Health Medical Center, Chongqing, China; ^2^ Infectious Diseases and Immunity in Global Health Program, Research Institute, McGill University Health Centre, Montréal, QC, Canada; ^3^ Chronic Viral Illness Service, McGill University Health Centre, Montréal, QC, Canada; ^4^ Canadian HIV Trials Network (CTN), Canadian Institutes of Health Research (CIHR), Vancouver, BC, Canada; ^5^ Division of Hematology, McGill University Health Centre, Montréal, QC, Canada

**Keywords:** HIV, alcohol, gut, microbial translocation, chronic inflammation

## Abstract

The intestinal microbiome is an essential so-called human “organ”, vital for the induction of innate immunity, for metabolizing nutrients, and for maintenance of the structural integrity of the intestinal barrier. HIV infection adversely influences the richness and diversity of the intestinal microbiome, resulting in structural and functional impairment of the intestinal barrier and an increased intestinal permeability. Pathogens and metabolites may thus cross the “leaky” intestinal barrier and enter the systemic circulation, which is a significant factor accounting for the persistent underlying chronic inflammatory state present in people living with HIV (PLWH). Additionally, alcohol use and abuse has been found to be prevalent in PLWH and has been strongly associated with the incidence and progression of HIV/AIDS. Recently, converging evidence has indicated that the mechanism underlying this phenomenon is related to intestinal microbiome and barrier function through numerous pathways. Alcohol acts as a “partner” with HIV in disrupting microbiome ecology, and thus impairing of the intestinal barrier. Optimizing the microbiome and restoring the integrity of the intestinal barrier is likely to be an effective adjunctive therapeutic strategy for PLWH. We herein critically review the interplay among HIV, alcohol, and the gut barrier, thus setting the scene with regards to development of effective strategies to counteract the dysregulated gut microbiome and the reduction of microbial translocation and inflammation in PLWH.

## Introduction

Although the widespread use of antiretroviral therapy (ART) has resulted in an increase in the lifespan of people living with HIV (PLWH), HIV/AIDS currently remains a major global public health problem (1). According to the Joint United Nations Programme on HIV/AIDS (UNAIDS) report, 690 000 people succumbed to AIDS-related diseases in 2019 (2). On the other hand, it is estimated that alcohol abuse causes 2.5 million deaths worldwide every year, and the World Health Organization lists alcohol consumption as an important risk factor for disease and disability worldwide (3).

Notably, alcohol use and abuse has been found to be highly prevalent in PLWH (4) and alcohol is strongly associated with the incidence and progression of HIV, including towards AIDS. Indeed, alcohol increases the risk of multiple comorbidities, such as hepatic fibrosis, hepatic cirrhosis, neurocognitive impairment, and AIDS-related dementia (HAD) (5–11). Moreover, alcohol abuse increases the risk of HIV infection by promoting risky behaviors (12). Alcohol abuse has also been associated with failure of ART to achieve virologic inhibition in PLWH (13–15).

Recent evidence highlights the fact that HIV and alcohol act as two “partners” in the disruption of the gut microbiota composition and impairment of the intestinal barrier, which leads to high-levels of microbial translocation and chronic immune activation in PLWH (16–18). The entire gastro-intestinal system hosts a large concentration and diversity of microbes. A population of nearly 100 trillion different microbes inhabits the human gut, comprising bacteria, archaea, fungi, yeasts, and viruses (19). Intestinal microbiota not only plays a vital role in maintaining the normal intestinal tract of individuals, but is also indispensable for the general health (20–22). Intestinal microbial dysbiosis, a disbalance of the gut microbiota composition, has been shown to be positively associated with several chronic conditions, such as obesity, cardiovascular disease, inflammatory bowel disease, cancer, and alcoholic liver disease (23, 24). HIV infection is known to be associated with microbial dysbiosis, intestinal barrier injury, and intestinal leakage (25). The “leaky gut” is now known to be one of the main factor causing the persistent underlying chronic inflammation in PLWH on ART, and is associated with poor recovery of CD4^+^ T-cell counts and the development and progression of non-AIDS-related conditions (26, 27). Moreover, alcohol use and abuse are known to enhance HIV-induced injury to the gut (16, 28). The present review will focus on the mechanisms whereby HIV and alcohol increase disruption to the gut microbiota and intestinal barrier, causing microbial translocation, and chronic systemic inflammation in PLWH. We will also discuss possible therapeutic strategies for the restoration of the structural and functional integrity of the intestinal barrier.

## Alcohol Use Accelerates the Progression of HIV Infection

Alcohol use is prevalent among PLWH around the world (4, 29–34). Due to the different population involved in alcohol use studies, the percentage of alcohol user varies in these studies. A study from Kampala, Uganda, observed that 33% of HIV-infected individuals self-reported any alcohol use, and 18.6% HIV-infected individuals were classified as alcohol abusers in 2012-2013 (33). Another study included 8567 HIV-infected individuals from the United States during 2013-2015, and showed that 41% of those were low alcohol users and 27% were hazardous alcohol users (34). The longitudinal cohort study by Kelso-Chichetto et al., found that among PLWH who were drinking alcohol, female were significantly less frequently found than male, and the percentage of heavy drinkers decreased in HIV-infected women during 10 years of follow-up; in contrast, the percentage of heavy drinkers in HIV-infected men who have sex with men (MSM) increased during follow-up (31). However, no consensus is currently accepted for the burden of alcohol use and abuse in PLWH (35, 36). Marshall et al. performed a longitudinal analysis among HIV-infected MSM, and found that the percentage of hazardous drinkers decreased from 29.0% to 24.2% during the eight-year follow-up (35). Moreover, a study in Porto Alegre, southern Brazil reported that heavy alcohol consumption among PLWH was even lower than the general population (5.6 vs. 10.3%) (36). The authors of the preceding study propose that the lower prevalence of risky alcohol consumption in PLWH may be secondary to their concern related to the perceived harmful consequences of alcohol use in negatively impacting HIV control (36). Importantly, PLWH experienced increased mortality and physiologic injury at lower levels of alcohol use compared with the general population (37).

The prevalence of alcohol use and abuse in PLWH is likely to induce tissue injury and reduce survival. Non-hazardous alcohol consumption once per week or more was reported to decrease survival in PLWH by 1 year, and by 6.4 years for those with daily hazardous consumption (38). In addition, alcohol consumption independently increases the risk for several comorbidities in PLWH, including the risk of dementia (8), cardiovascular disease (39, 40), hepatic cirrhosis (41), and pneumonia (42). A study by Freiberg et al., showed that compared with infrequent and moderate drinking, hazardous drinking and alcohol abuse were associated with a higher prevalence of cardiovascular diseases (39). Moreover, liver injury is known to be a major cause of morbidity and mortality among PLWH (43, 44). Alcohol is a potent trigger of HIV-mediated liver damage, which accelerates hepatic disease progression and eventually results in advanced fibrosis and cirrhosis (7, 45). A probable mechanism for liver inflammation and fibrosis was proposed by Chen et al. (46): alcohol increases intestinal permeability and gut-derived pathogens cross the intestinal barrier to enter into the liver, then hepatic stellate cells, Kupffer cells, and hepatocytes are activated to secrete pro-inflammatory cytokines and chemokines, causing persistent inflammation and injury to the liver (46). Alcohol may also promote HIV-mediated liver injury through increased oxidative stress and mitochondrial disorders, leading to increased virus replication and hepatocyte apoptosis (41, 44, 47–51). Reports have shown that alcohol use can activate microglial cells and astrocytes, promoting neuronal cell death by enhancing oxidative stress and gut microbiome changes, eventually leading to impaired cognition and behavioral deficits, and possibly death (8, 52–55).

Alcohol modulates immune cells and increases systemic inflammation, which has been considered to be one of the main mechanisms for adverse outcomes induced by alcohol use and abuse. In simian immunodeficiency virus (SIV)-infected rhesus macaques, alcohol use has been shown to accelerate the decline of peripheral CD4^+^ T-cells (56). However, the results of related observational studies in PLWH in Uganda have been conflicting, indicating conversely, that unhealthy alcohol use may not accelerate CD4^+^ T-cell decline in PLWH (57). Alcohol use is also reported to alter CD8^+^ T-cell phenotypes in PLWH, and alcohol is positively associated with activated-senescent and terminal effector memory CD45RA^+^CD8^+^ T-cells, but not CD4^+^ T-cells (17). In addition, alcohol use additively or synergistically increases systemic inflammatory factors in PLWH. A study of HIV-infected individuals in Russia showed that alcohol use and abuse independently increased levels of the following biomarkers: soluble CD14 (sCD14), interleukin (IL)-6, and D-dimer (58). Monnig et al. reported that HIV-infected individuals had higher levels of lipopolysaccharide (LPS), LPS-binding protein (LBP), sCD14, and soluble CD163 (sCD163) than uninfected individuals with similar alcohol use (59). Of note, these biomarkers have been associated with increased mortality risk in PLWH (60–62).

Moreover, alcohol use and abuse in PLWH has become an important factor in reducing adherence to ART, leading to poor ART efficacy (63–66), and increasing the possibility of antiretroviral drug resistance (67, 68). An epidemiological study of HIV-infected women on ART by Howard et al., illustrated the relationship between antiretroviral adherence and viral load. Virological failure occurred in 17% of women with adherence rates of greater than or equal to 88%, in 28% of those with 45-87% adherence, in 43% of those with 13-44% adherence, and in 71% of those with less than or equal to 12% adherence (69). Alcohol use was a significant predictor of lower adherence (70, 71), and in an investigation by Braithwaite and colleagues, they observed that regardless of HIV status and temporal and dose-response relationships between alcohol consumption and missed HIV medications, consumption of alcohol was associated with decreased adherence to medications on that day and on the following two days. In particular, among non-binge drinkers (i.e., drinkers who consumed less than five standard drinks per day), 3.5% missed medication doses on drinking days, 3.1% missed medication on post-drinking days, and 2.1% missed medication on non-drinking days (*p*<0.001 for trend). Among binge drinkers (i.e., drinkers who consumed five or more drinks per day), 11.0% missed doses on drinking days, 7.0% missed medication on post-drinking days, and 4.1% missed medication on non-drinking days (*p*<0.001 for trend) (72).

Furthermore, alcohol may aggravate the toxicity of ART drugs, which is likely to decrease ART adherence (65). Hepatoxicity is one of most common side effects for ART drugs. In the liver, the main metabolic pathway for the metabolism of alcohol as well as antiretroviral drugs (such as zidovudine, stavudine, and nevirapine) is the cytochrome P450 pathway; thus alcohol use may aggravate the adverse effects of antiretroviral drugs *via* competitive inhibition of the cytochrome P450 pathway (7, 73). In addition, alcohol may increase the adverse effects of ART drugs on testicular function (74). Moreover, beliefs that mixing alcohol and ART drugs is toxic, and that drug treatments should be interrupted when drinking are common among PLWH, thus also leading to treatment nonadherence (4).

Aside from poor adherence to ART caused by alcohol, increased viral replication induced by alcohol is a further potential reason for ART failure. In HIV-infected peripheral blood lymphocytes (PBLs) pretreated with alcohol, HIV-1 DNA increased 10-fold, and it has been observed that alcohol enhanced the expression of the chemokine receptor 4 (CXCR4) HIV-entry co-receptor (75). Two studies of chronic alcohol consumption in rhesus macaques observed similar results, with the plasma viral load in the alcohol group being much higher than that in the control group (76, 77).

## HIV Infection Is Associated With Gut Microbiome Dysbiosis and Related Inflammation

The gut contains a large proportion of lymphoid tissue and lymphocytes of the human body (78, 79), and is one of the earliest targets of, and a reservoir for, HIV infection (80). HIV directly attacks the gut mucosal epithelium, leading to intercellular tight junction disruption and death of enterocytes, increasing gut permeability (26, 81, 82). Primary gut mucosal CD4^+^ T-cells have higher chemokine receptor 5 (CCR5) co-receptor expression than peripheral blood CD4^+^ T-cells (83–85). Thus, gut CD4^+^ T-cells are priority targets for HIV, support higher levels of viral replication (85, 86), and are massively depleted during early HIV infection (87). One study reported that CD4^+^ T-cell depletion occurs during all stages of HIV disease, and this occurs predominantly in the gastrointestinal tract (GI) (87). Overall, HIV infection leads to intestinal epithelial damage and a reduction in numbers of immune cells.

**Figure 1:**
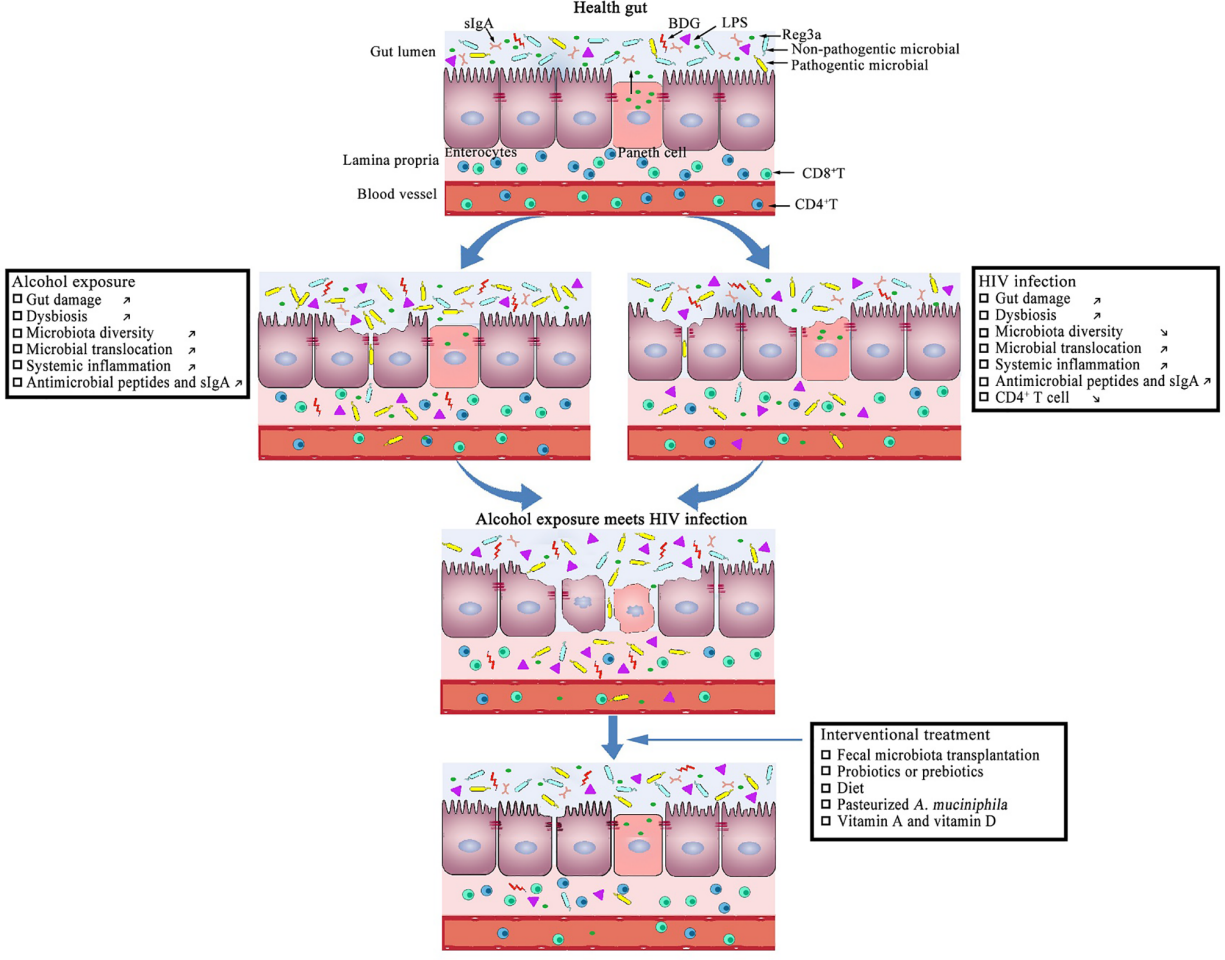
Potential effects of alcohol exposure and HIV infection on intestinal mucosal integrity.

Recently, a number of studies have indicated that HIV infection is associated with intestinal microbial dysbiosis (**Figure 1**). HIV infection significantly affects gut microbial richness and diversity (26, 81, 88–90), especially in immune discordant patients (91). Lozupone et al. reported that HIV infection may reduce abundance of the symbiotic bacterium *Bacteroides fragilis* by depleting gut CD4^+^ T-cells (92). Vujkovic-Cvijin et al. reported that compared with HIV-negative individuals, the gut microbial communities of HIV^+^ viremic untreated individuals is mainly altered by an increased abundance of Proteobacteria and Bacteroidaceae; however, effective ART fails to completely reverse these changes (93). In addition, several studies have shown that enrichment in the bacterial genus *Prevotella* and a depletion of *Bacteroides* occurs in untreated PLWH (92, 94, 95). Compared with HIV^+^ viremic progressors, the genera *Succinivibrio*, *Sutterella*, *Rhizobium*, *Delftia*, *Anaerofilum*, and *Oscillospira* were more abundant in elite controllers, whereas the genera *Blautia* and *Anaerostipes* were depleted (96). Rocafort et al., reported that HIV infection reduces the abundance of *Akkermansia*, *Anaerovibrio*, *Bifidobacterium*, and *Clostridium* (97). Besides microbial compositions, HIV infection also causes changes to microbial and cellular metabolites. HIV infection and microbial translocation have been linked to increased catabolism of tryptophan into kynurenine. Indoleamine 2,3-dioxygenase 1 (IDO-1) is the main inducible and rate-limiting enzyme for the catabolism of tryptophan through the kynurenine pathway (93, 98), and is up-regulated by interferons (IFNs) and by agonists of Toll-like receptors (TLRs) (99). In PLWH, IDO-1 activity is increased, and this is thought to be related to plasma levels of LPS and (1, 3)-β-D-Glucan (BDG), Treg cell frequency, microbial translocation, immune activation, and HIV reservoir size (93, 100–103). Moreover, the study by Vujkovic-Cvijin et al. showed that HIV infection-related intestinal microbiota participate in tryptophan metabolism, compared with the intestinal microbiota of HIV-negative individuals. HIV-positive viremic, untreated individuals had an enrichment of more genetic homologs to tryptophan catabolism enzymes of the kynurenine pathway in their intestinal microbiota, and that, perhaps, contributes to immunoactive tryptophan catabolism during HIV disease (93). Trimethylamine-N-oxide (TMAO) is an intestinal microbiota-dependent metabolite of phosphatidylcholine (104), and a strong relationship between TMAO and increased risk for atherosclerosis and cardiovascular disease has been reported (105, 106). Shan et al. reported that plasma TMAO levels positively correlate with serum biomarkers of monocyte activation and inflammation, and is associated with progression of carotid atherosclerosis in PLWH (107). Butyrate, one of the most abundant short-chain fatty acids (SCFA) in the intestinal tract, provides the primary energy source for epithelial colonic cells, promotes epithelial barrier integrity, prevents microbial translocation, and further reduces inflammation (108–110). Compared with HIV-negative individuals, a number of the bacterial genera associated with producing butyrate (e.g., *Roseburia*, *Coprococcus*, *Faecalibacterium prausnitzii*, and *Eubacterium rectale*) are less frequent in HIV-positive individuals (110–112). Moreover, a low abundance of butyrate-producing bacteria in the colon is reported to be associated with microbial translocation and immune activation in PLWH (110). Furthermore, evidence has shown that gut damage and dysbiosis induce higher levels of microbial translocation. One study by Raffatellu et al., observed that after eight hours, SIV-infected macaques had significantly higher levels of *Salmonella typhimurium* in the mesenteric lymph nodes than SIV-negative macaques, subsequent to injection of *S. typhimurium* into the gut lumen (113). Estes et al. using quantitative image analysis, revealed that damaged intestinal epithelium was associated with microbial translocation in SIV-infected macaques (81).

Gut microbial translocation resulting from dysbiosis and gut damage plays a prominent role in maintaining a persistent underlying chronic inflammatory state in PLWH, and compliant, long-term ART does not entirely reverse damage to the intestinal tract barrier (81, 90, 114–117). Thus the gut fails to successfully repair in PLWH receiving ART (90, 114, 115). Measurement of specific plasma biomarkers is a convenient way to assess the level of gut damage and microbial translocation, as endoscopy remains difficult (118–121). LPS is a component of the cell wall of Gram-negative bacteria, and is well-known to stimulate innate and adaptive immunity *in vivo* (90), Marchetti et al., analyzed 1488 biomarker measures from 379 HIV-infected individuals, and observed that LPS was an effective biomarker associated with accelerated disease progression independently of age, HIV RNA loads, and CD4^+^ T-cell counts (122). Moreover, compared with immunological responders, higher LPS levels were detected in immunological non-responders (INRs), and the higher LPS levels in INRs were associated with impairment of CD4^+^ T-cell reconstitution by sustaining T-cell hyperactivation (123). BDG is a component of the cell wall of fungi, and identification of plasma BDG is currently used for the clinical diagnosis of invasive fungal infections (124). Morris et al. reported that high serum levels of BDG are associated with a decrease of CD4^+^ T-cell counts, a higher viral load, and activation of CD8^+^ T-cells in PLWH (125, 126). Intestinal fatty acid binding protein (I-FABP), expressed in enterocytes, is released upon cell death, and enters into the systemic circulation (127). HIV infection increases plasma levels of I-FABP in PLWH (128, 129), but sustained effective ART has not been shown to completely reverse these levels in plasma (130). Regenerating islet-derived protein 3-α (REG3α) is an antimicrobial peptide secreted by Paneth cells into the gut lumen, and translocates into the blood when the integrity of the intestinal epithelium is compromised (131). REG3α levels are higher in PLWH, and are associated with lower CD4^+^ T-cell counts and CD4/CD8 ratios, which positively correlate with HIV disease progression (131). Thus, increased microbial translocation in HIV-infected individuals is likely to contribute to persisting inflammation and disease progression in PLWH.

## Alcohol Use Causes Disruption of the Intestinal Barrier

The function of the intestinal barrier is to regulate the absorption of water and key nutrients from the gut lumen into the bloodstream, and to prevent pro-inflammatory microbial products from entering into the portal and systemic circulation (132). Intestinal barrier disruption, also referred to as “intestinal leakiness”, results in increasing intestinal permeability, thus permitting the passage of pathogens and microbial products into the bloodstream (133–135). As shown in **Figure 1**, many studies have indicated that alcohol use disrupts the intestinal barrier and increases intestinal permeability (136–138). Leclercq et al., measured intestinal permeability using an oral stable, non-degradable radioactive chromium-51 probe in the body, called ^51^Cr-EDTA, and by examining the resulting radioactivity in urine. Their results showed that compared with non-alcohol-user subjects, intestinal permeability was largely increased in alcohol-dependent subjects (139). Tang et al. observed comparable results, showing that chronic alcohol consumption increased intestinal permeability in mice (138).

Several mechanisms have been reported to be associated with the alcohol-induced intestinal disruption. Alcohol and its metabolites damage enterocytes and villi tips directly, and weaken cell membranes by the generation of reactive oxygen species (ROS) released during alcohol metabolism, thus allowing material such as LPS, alcohol, and microbial products to pass directly through the epithelial cells (133, 140, 141). Also, alcohol disrupts intestinal epithelial cellular integrity by inducing enterocytic apoptosis (142) and an intestinal stem cell decrease in frequency (143). Additionally, alcohol reduces expression of intestinal tight junction and adherent junction proteins in enterocytes, which causes disruption of intercellular junctions (142, 144, 145). Ren et al. reported that the down-regulated expression of tight junction proteins in alcohol treated Caco-2 cells activated the tumor necrosis factor alpha (TNF-α) and nuclear factor kappa-B (NF-κB) signaling pathways (146). Moreover, alcohol can cause overexpression of microRNA (miRNA), such as miR-155, miR-122, and miR-212 in the intestine, which may also affect the gut barrier by regulating genes associated with intestinal mucosal cell integrity (147–149).

Studies have also observed that alcohol directly modulates intestinal innate and adaptive immune responses, resulting in modulation on clearance of pathogens and gut-derived inflammation. Alcohol inhibits the intestine’s immune response for clearing *S. typhimurium* in the gut (150). An early study by Lopez et al. showed the effect of chronic alcohol exposure on intestinal Peyer’s patches (PPs), sites where naive immune cells differentiate into a variety of mature immune cell subsets (151). Compared with a non-exposed mouse model, a significant decrease in the total number of cells was observed in the PPs of mice exposed to alcohol for 5 weeks, and a highly significant decrease was observed in mice exposed to alcohol for 19 weeks (151). Similarly, a study in mice showed that chronic alcohol consumption decreased the proportion of T-cells, induced alterations in dendritic cells and macrophages in the intestine, and decreased the levels of IgA in small intestinal fecal contents (152). Furthermore, alcohol also induces the downregulation of the host antimicrobial peptides regenerating islet-derived protein 3-ß (REG3ß) and regenerating islet-derived protein 3-γ (REG3γ), which resulted in bacterial overgrowth (153). The preceding studies are important in demonstrating that alcohol use adversely affects mucosal immune mechanisms.

Alcohol consumption also causes bacterial overgrowth and dysbiosis in animals and humans (154–157), and alcohol-related microbial products have been reported to contribute to increased intestinal permeability and peripheral immune activation (158). Alcohol-treated mice had both aerobic and anaerobic bacteria more frequently present in the proximal small intestine, compared with control mice fed with an isocaloric liquid diet. Also, frequency of Bacteroidetes and Verrucomicrobia bacteria were up-regulated in alcohol receiving mice. REG3ß and REG3γ genes were down-regulated in alcohol-fed mice (157). In humans, small intestine bacterial overgrowth is closely associated with the severity of alcoholic cirrhosis (159), and is a risk factor for hepatic encephalopathy (160). Chen et al. reported that the abundance of Prevotellaceae, Enterobacteriaceae, Veillonellaceae, and Streptococcaceae was significantly increased in alcoholic cirrhotic patients, compared with control subjects (161). Engen et al. summarized the changes in gut microbial communities in alcoholics and cirrhotic patients, where microbial communities Gammaproteobacteria and Bacilli firmicutes were enriched. In contrast, firmicutes of the class Clostridia were depleted in alcoholics but were not significantly altered in the cirrhotic group (162). Moreover, alcohol consumption has been shown to be associated changes in levels of microbial metabolites, including SCFA, long-chain fatty acids (LCFA), bile acids and tryptophan (163–166). Several investigations have indicated that alcohol decreased SCFA and LCFA levels in a murine model of chronic alcohol consumption, and supplementation of SCFA and LCFA reduces alcohol-induced liver injury (163, 164, 167). Hendrikx et al., reported that alcohol interfered with tryptophan catabolism, and decreased indole-3-acetic acid, resulting in a decreased expression of IL-22 in the intestine and reduced the expression of antimicrobial peptide REG3γ (165). Xie et al. reported that chronic alcohol consumption led to increased expression of genes involved in bile acid biosynthesis and efflux transport in the liver, and observed that taurine-conjugated bile acids were significantly decreased, and unconjugated and glycine-conjugated bile acids increased in the GI of alcohol-treated rats (166). Interestingly, antibiotics have been found to abrogate intestinal bacterial overgrowth and alleviate subsequent liver damage in rodents (168). Several studies have found that probiotics promote growth of beneficial bacteria, alleviate alcoholic liver injury in rats (169), and improve alcohol-associated liver dysfunction in cirrhotic patients (170, 171).

## The Combined Effects of HIV and Alcohol on the Intestinal Barrier

Current evidence indicates that HIV and alcohol could have a deleterious synergistic effect in the gut. In a murine model, HIV transgenic rats are more susceptible to alcohol-induced gut leakiness, hepatic steatosis and inflammation than the corresponding wild-type rats (172, 173). Samuelson et al. observed that alcohol-associated intestinal dysbiosis mediated the susceptibility to pneumococcal pneumonia in a humanized mouse HIV model (174). In concordance with the animal model, Webel et al. found that alcohol consumption was associated with a range of markers of gut permeability, microbial translocation, immune activation, and inflammation in ART-treated PLWH (16). Maffei et al. reported that alcohol use is associated with a dysfunctional CD8^+^ T-Cell phenotype, intestinal leakiness, and dysbiosis among PLWH (17). As far as can currently be ascertained, the potential interactive mechanisms between HIV and alcohol in the GI tract has not yet been well elucidated. However, in view of the known individual effects of HIV and alcohol, we speculate that they (HIV and alcohol) may together exhibit additive or synergistic interactions causing disruption to microbial ecology and impairment of the intestinal barrier through several pathways.

The changes relating to dysbiosis in gut microbiota composition observed in past studies with respect to the effects of alcohol and HIV have not always been found to be consistent, as different studies have involved different populations and these studies were conducted in varying disease contexts. Most studies have shown that both alcohol and HIV can indeed induce the dysbiosis involved with decreased frequency of “beneficial” microorganisms and enrichment of “harmful” pathogens. Specifically, the beneficial bacteria *Bifidobacteria*, *Lactobacillus*, and *Akkermansia muciniphila* were decreased, while *Candida albicans* was increased in PLWH and in individuals using alcohol (97, 123, 175–178).

Moreover, dysregulation of the gut microbiota metabolism induced by alcohol and HIV may also play a vital role in the disruption of microbial ecology and impairment of the intestinal barrier. In PLWH, there is a lower abundance of butyrate-producing bacteria and butyric acid levels in feces (179). It was also been observed that butyric acid was significantly reduced in colonic and rectal contents in a rat model of chronic alcohol consumption (163). Moreover, in PLWH, the dysbiosis was associated with increased catabolism of tryptophan into kynurenine and resulting in intestinal barrier destruction (93, 180). It has also been reported that alcohol perturbed tryptophan catabolism, decreased indole-3-acetic acid, resulting in a decreased expression of IL-22 in the intestine and a reduction of the expression of the antimicrobial peptide REG3γ (165).

Other factors, including apoptosis and oxidative stress of intestinal epithelial cells, and intestinal tight junction and adherent junction protein dysfunction may contribute to their synergistic effects. Indeed, both alcohol and HIV could promote apoptosis of intestinal epithelial cells (142, 181), increase oxidative stress of cells (133, 182) and decrease the expression of intestinal tight junction and adherent junction proteins (26, 142, 143). Overall, these factors are likely to work together to promote gut permeability, enhance microbial translocation, and increase gut and systemic inflammatory responses, further contributing to an increased risk of non-AIDS comorbidities in PLWH. However, the questions as to precisely in what manner alcohol and HIV interact with each other on the disruption of the gut homeostasis, and precisely which factors play the most critical roles in negatively impacting on the intestinal barrier when alcohol and HIV are simultaneously present in the gut, remain to be answered. Future further investigations are warranted to discover coherent answers to these important questions.

## Therapeutic Strategies to Improve the Intestinal Barrier

In view of the significant structural and functional changes caused by HIV infection combined with alcohol use to the intestinal epithelial barrier, attempts at optimization of the microbiota and restoration of intestinal barrier function may be an effective adjunctive treatment option in PLWH. Numerous microbiota-based therapies have been studied in the condition of HIV infection and alcohol consumption (**Table 1**). Aside from these strategies, multiple clinical trials using various interventions are ongoing in individuals with conditions (**Table 2**).

**Table 1:** Microbiome-based therapies in the context of HIV infection and alcohol consumption.

Study	Model	Intervention	Design	Change of fecal microbiota by experimental intervention	Change of cytokines and immune cells by experimental intervention
**HIV**					
Gori et al. 2011 (183)	ART-naive HIV-infected patients (57 cases)	Arm I: 15 or 30 g short chain galactooligosaccharides/long chain fructooligosaccharides/pectin hydrolysate-derived acidic oligosaccharides Arm II: 30 g short chain galactooligosaccharides/long chain fructooligosaccharides/pectin hydrolysate-derived acidic oligosaccharides Arm III: placebo	Double-blinded, randomized	Increase: bifidobacterial Decrease: Clostridium coccoides/Eubacterium rectale cluster, Clostridium perfringens and Clostridium difficile species	Increase: NK cells Decrease: sCD14, activated CD4^+^/CD25^+^ T cells
Stiksrud et al., 2015 (184)	HIV-infected ART-suppressed individuals (24 cases)	Arm I: 250 mL/d fermented skimmed milk supplemented with Lactobacillus rhamnosus GG (10^8^ cfu/mL), Bifidobacterium animalis subsp. lactis B-12 (10^8^ cfu/mL), and Lactobacillus acidophilus cfu/mL) Arm II: placebo	Double-blinded, randomized	Increase: Bifidobacteria and Lactobacilli Decrease: Bacteroides	Decrease: D-dimer, C-reactive protein, IL-6 No significant changes in T-cell activation
Hensley-McBain et al., 2016 (185)	Rhesus macaque (SIV)-infected macaques (6 cases)	Arm I: FMT(Stool samples from healthy (SIV-negative) rhesus macaque donors)	Open-label	The microbiome composition quickly reverted by 2 weeks, similar to the findings pre-transplantation	No significant difference in LPS, IL-6, CRP.
Vujkovic-Cvijin et al., 2017 (186)	HIV-infected ART-suppressed individuals (8 cases)	FMT (low abundance of Proteobacteria and high abundance of Bacteroidetes)	Open-label, randomized	Increase: Faecalibacterium and Rikenellaceae family. Decreases Erysipelotrichaceae family	No significant difference in IL-6, sCD 14, HLA-DR CD8^+^ T cells
Sainz et al., 2020 (187)	HIV-infected children (22 cases)	Arm I: long chain fructo-oligosaccharides, galacto-oligosaccharides, Saccharomyces boulardii, essential amino acids arginine, glutamine, vitamin D and AM3 Arm II: placebo	Double-blinded, randomized	Increase: in Prevotella, Akkermansia and Escherichia Decrease: in commensals Faecalibacterium	N/A
Serrano-Villar et al., 2021 (188)	ART- HIV-infected patients (29 cases)	Arm I: FMT (enrichment for Bacteroides and Faecalibacterium genus and depletion of Prevotella genus) Arm II: placebo	Double-blinded, randomized	Increase: *Anaerostipes* spp., *Blautia* spp., *Dorea* spp., and *Fusicatenibacter* spp.	Decrease: I-FABP No significant difference in circulating CD4^+^, CD8^+^ T cells, and the CD4/CD8 ratio
**alcohol**					
Kirpich et al., 2008 (189)	Alcoholic male patients (66 cases)	Arm I: vitamin B1 and B6 Arm II: vitamin B1 and B6, and supplement with 0.9 × 10^8^ CFU Bifidobacterium bifidum and 0.9 × 10^9^ CFU Lactobacillus plantarum 8PA3	Open label, randomized	Increase: *bifidobacteria* and *lactobacilli*	Decrease: AST, ALT, LDH and total bilirubin
Stadlbauer et al., 2008 (190)	Patients with alcoholic cirrhosis (20 cases)	Arm I: Lactobacillus casei Shirota (6.5 × 10^9^ cfu/mL)	Open label	N/A	Increase: TLR4 Decrease: IL-10
Philips et al., 2018 (191)	Alcoholic liver patients (16 cases)	Arm I: FMT(stool samples from healthy donors)	Open-label	Increase: *Roseburia* and *Micrococcus*	N/A
Macnaughtan et al., 2020 (192)	Alcoholic cirrhosis patients (92 cases)	Arm I: probiotic Lactobacillus casei Shirota(6.5 × 10^9^ cfu/bottle) Arm II: placebo	Double-blinded, randomized	N/A	Significantly reduced plasma monocyte chemotactic protein-1,IL-1β, IL-17a and macrophage inflammatory protein-1β
Wang et al., 2021 (193)	Alcoholic liver mice (42 cases)	Arm I: 0.9% normal saline Arm II: white spirit Arm III: white spirit and the Fermentation broth of the mixture of pueraria lobata, lonicera japonica, and crataegus pinnatifida by Lactobacillus rhamnosus 217-1	Open-label	Increase: *Lachnospiraceae* and *Lactobacillus*	Increase: superoxide dismutase, and glutathione Decrease: AST, LPS, IL-6, TNF-ɑ

N/A, not available; AST, aspartate aminotransferase; ALT, alanine aminotransferase; LDH, lactate dehydrogenase; TLR4, toll like receptor 4; CFU, colony forming units.

**Table 2:** Ongoing clinical trials on reduction of microbial translocation and restoration of the intestinal barrier.

Clinical trial number	Condition	Design	Objectives	Intervention	Number of participants	Locations
NCT01466595	HIV-1 infection	RCT	Rifaximin modulate gut microbial translocation and systemic immune activation	ARM I: rifaximin ARM II: placebo	73	US
NCT01839734	HIV-1 infection	RCT	Lubiprostone modulate gut microbial translocation and systemic immune activation	ARM I: lubiprostone ARM II: placebo	20	US
NCT02431325	HIV-1 infection	RCT	Investigate teduglutide repair gut barrier, decrease inflammation	ARM I: teduglutide ARM II: placebo	50	US
NCT02164344	HIV-1 infection	RCT	Effects of probiotics on microbial translocation and immune activation	Dietary supplement: probiotics	30	US
NCT04111263	Gastrointestinal Injury, Acute Mountain Sickness	RCT	Nutritional intervention for gut barrier integrity at high altitude	ARM I: placebo and high altitude exposure ARM II: fiber and polyphenol supplementation and high altitude exposure ARM III: placebo and sea level exposure	15	US
NCT01877044	Obesity, Overweight	RCT	Long-term effects of arabinoxylans on intestinal barrier function	ARM I: arabinoxylans ARM II: placebo	46	Netherlands
NCT01792388	Crohn’s Disease	RCT	Vitamin D improve barrier function in IBD	ARM I: vitamin D ARM II: placebo	30	Ireland
NCT02862249	Liver Cirrhosis	RCT	Assess whether restoring gut microbiota with FMT in patients with advanced cirrhosis is both feasible and safe	ARM I: FMT under gastroscopy ARM II: placebo under gastroscopy	32	UK
NCT03482284	Healthy subjects	RCT	the effect of monosaccharides on intestinal barrier function	Dietary supplement: monosaccharide	12	Austria
NCT04598295	Irritable Bowel Syndrome	RCT	Assess the gastrointestinal symptomatic impact of DS-01 on IBS	ARM I: DS-01(include 24-beneficial strains) ARM II: placebo	100	US
NCT02875847	Irritable Bowel Syndrome	RCT	Establish the effect of HMOs on the fecal microbiota in IBS patients	ARM I: HMO1 ARM II: HMO2 ARM III: placebo	60	Sweden
NCT03973996	Endotoxemia	RCT	Examine the efficacy of green tea on metabolic endotoxemia	ARM I: green tea ARM II: placebo	40	US
NCT03791866	Sepsis	RCT	Investigate the mechanisms of early enteral nutrition (EEN) maintaining intestinal mucosal barrier in sepsis	ARM I: 30% target total enteral nutrition ARM II: 60% target total enteral nutrition ARM III: 100% target total enteral nutrition	60	China

IBD, Inflammatory bowel disease; IBS, Irritable bowel syndrome; FMT, Fecal microbiota transplantation; RCT, Randomized controlled trial.

Probiotics and prebiotics have been considered to be an effective adjunctive treatment strategy in PLWH. A study by d’Ettorre et al., showed that when oral probiotics (with an abundance of *Streptococcus salivarius* and *Bifidobacteria*) were administered together with ART to PLWH, subjects showed a decrease in CD4^+^ T-cell activation, and lower levels of sCD14, lipopolysaccharide binding protein (LBP), and C-reactive protein (CRP) compared with the control group (194). Serrano-Villar et al. reported that use of oral prebiotics (including short-chain galacto-oligosaccharides, long-chain fructo-oligosaccharides, and glutamine) in PLWH may reduce levels of IL-6, CRP, D-dimer, and T-cell activation, and increase thymic output. In addition, it has been observed in PLWH that prebiotics increased the abundance of *Faecalibacterium* and *Lachnospira*, which strongly correlated with a significant increase in butyrate production and a decrease of inflammatory biomarkers sCD14 and CRP (195).

*Akkermansia muciniphila*, an anaerobic symbiotic bacterium, was reported to increase the thickness of mucus, improve enterocyte monolayer integrity, and counteract inflammation (196–199). It has been reported that the abundance of *A. muciniphila* is significantly decreased in inflammatory bowel disease (IBD), and supplementation of *A. muciniphila* in the gut improved colitis by decreasing colon-infiltrating macrophages and cytotoxic T-lymphocytes (CTLs) (200). Moreover, HIV infection and alcohol consumption induced *A. muciniphila* depletion in the intestine (97, 201). Thus, increasing the abundance of *A. muciniphila* in the intestine seems to be an effective therapeutic strategy to restore the integrity of the intestinal barrier. An alternative strategy to increase the abundance of *A. muciniphila* in the gut is by consumption of concord grapes, cranberries, and the camu camu fruit (202). The commonly-used therapeutic drugs metformin and vancomycin have also been reported to increase the abundance of *A. muciniphila* in the intestinal tract (202, 203).

Fecal microbiota transplantation (FMT) refers to transplantation of fecal microbes from a healthy donor to the GI of a recipient, and is currently being utilized to enhance gut microbial diversity. A large body of evidence has shown that FMT is a highly effective treatment modality against *Clostridium difficile* infection, and the levels of pro-inflammatory cytokines (TNF-α, IL-1β, IL-6, IL-8 and IL-12) significantly decreased after FMT (204–207). In addition, FMT was reported to restore graft-vs.-host disease (GVHD)-induced intestinal dysbiosis, as reported by Spindelboeck et al., in three severe acute GVHD patients. The restoration of a significantly more diverse microbiome was observed after one to six FMTs delivered *via* colonoscopy (206). In PLWH and animal models, FMT may be a viable method to restore the intestinal barrier. One study by Hensley-McBain et al., demonstrated that increased peripheral CD4^+^ T helper (Th)-17 and -22 frequencies and decreased gut CD4^+^ T-cell activation occurs after transplantation of healthy (SIV-negative) rhesus macaque fecal matter to SIV-infected rhesus macaques (185). A pilot study by Vujkovic-Cvijin et al., showed one-time FMT was well-tolerated in ART-treated PLWH and could lead to partial microbial engraftment including an increase of Faecalibacterium (208), which has exhibited anti-inflammatory effects in cellular and animal models (209). In addition, Serrano-Villar et al. reported that repeated oral FMT capsules caused long-lasting effects in the recipients’ microbiome, specifically in several members of the Lachnospiraceae family. A significant amelioration of the gut damage biomarker I-FABP was also observed in the FMT group (188).

Other strategies to restore intestinal function exist. For example, there may be a role for IL-22-secreting T-cell populations in limiting microbial translocation and systemic inflammation (25). Supplementation of IL-22 may be an effective treatment, and local IL-22 gene delivery improves intestinal inflammation by enhanced signal transducer and activator of transcription 3 (STAT3) activation within colonic epithelial cells in the murine model of ulcerative colitis (210). Studies by Hendrikx et al. observed that feeding mice engineered bacteria that produce IL-22 increased the expression of small intestinal Reg3γ and reduced microbial translocation (165). Furthermore, vitamin A and vitamin D are also known to play a role in maintaining intestinal function. Vitamin A and vitamin D regulate the tight junction protein expression of intestinal tight junction protein 1 (ZO-1), Occludin, and Claudin. In addition, the maturation of group 3 innate lymphoid cells (ILC3) that produce IL-22 and Treg cells that produce IL-10 also requires vitamin A and vitamin D. Interestingly, alcohol consumption was reported to reduce vitamin A and vitamin D circulating levels (211, 212). Supplementation of vitamin A and/or vitamin D may be a potential therapeutic strategy to restore a structurally and functionally intact intestinal barrier (213). The combination of IL-21 and probiotic therapy increases Th17 cell counts and decreases the marker for microbial translocation in ART-treated, SIV-infected rhesus macaques (214). Recombinant human IL-7 increases both circulating and gut-residing naïve and memory CD4^+^ T-cells, and decreases plasma levels of sCD14 and D-dimer in HIV-infected individuals (215, 216). Finally, Mallarino-Haeger et al. reported that the usage of dipyridamole, a blood vessel dilator, in ART-treated PLWH can significantly increase extracellular adenosine levels, minorly reduce plasma I-FABP levels, and affect regulation of gut mucosal immunity (217).

## Conclusion

In summary, in this review we have endeavored to highlight the associations between HIV infection, alcohol use and abuse and gastrointestinal injury in PLWH. Alcohol use and abuse leads to the failure of ART and reduces the survival time of HIV-infected individuals. The intestine is an immunologically indispensable organ, which is structurally and functionally impaired by alcohol use in PLWH. Among HIV-infected individuals, alcohol use further increases intestinal permeability, negatively affects the richness and diversity of the intestinal microbiota, and promotes microbial translocation, chronic immune activation, and chronic inflammation. The resultant underlying state of chronic inflammation increases the risk of development of further comorbidities and disease progression. Several studies have shown that changes in diet and enhancements of the diversity of intestinal microbiota may help reduce intestinal immune activation and subsequent chronic inflammation. Further investigation is warranted in order to study and elucidate the roles of intestinal bacteria and fungi in host immune defense mechanisms, and to explore new potential therapeutic strategies for the effective enhancement of host intestinal immune function, including in the context of alcohol use in PLWH or other conditions.

## Author Contributions

JY and JO wrote the first draft of the manuscript. SI, XZ, and VJ provided critical revision of the manuscript. J-PR and YC conceived and designed the manuscript. All authors read and approved the final manuscript.

## Funding

This work was supported by the Joint Medical Research Project (2020GDRC010) of Chongqing Science & Technology Bureau and Chongqing Health Commission, Chinese Federation of Public Health foundation (GWLM202024) and Chongqing Talent Cultivation Program (cstc2021ycjh-bgzxm0275).

## Conflict of Interest

The authors declare that the research was conducted in the absence of any commercial or financial relationships that could be construed as a potential conflict of interest.

## Publisher’s Note

All claims expressed in this article are solely those of the authors and do not necessarily represent those of their affiliated organizations, or those of the publisher, the editors and the reviewers. Any product that may be evaluated in this article, or claim that may be made by its manufacturer, is not guaranteed or endorsed by the publisher.
